# Idiopathic myointimal hyperplasia of the mesenteric veins: Case report and review of the literature

**DOI:** 10.1002/jgh3.12297

**Published:** 2019-12-27

**Authors:** Felicity C Martin, Linda S Yang, Sasha R Fehily, Basil D'Souza, Allan Lim, Penelope A McKelvie

**Affiliations:** ^1^ Department of Gastroenterology St Vincent's Hospital Fitzroy Victoria Australia; ^2^ Department of Colorectal Surgery St Vincent's Hospital Fitzroy Victoria Australia; ^3^ Department of Pathology St Vincent's Hospital Fitzroy Victoria Australia

**Keywords:** case report, colitis, idiopathic myointimal hyperplasia of mesenteric veins, ischemia, review

## Abstract

In 1991, Genta and Haggitt described four patients with segmental ischemic colitis caused by idiopathic myointimal hyperplasia in the small mesenteric veins (IMHMV). There are now 33 published cases of IMHMV in the literature; however, this condition is still sufficiently rare that it poses a diagnostic challenge to pathologists and clinicians and is often clinically or histologically confused with inflammatory bowel disease (IBD) or ischemic colitis. IMHMV is characterized by intimal smooth muscle hyperplasia resulting in thickened small and medium‐sized mesenteric veins (with arterial sparing). Clinically, it presents with symptoms that mimic IBD, such as bloody diarrhea, abdominal pain, and weight loss. Surgical resection appears to be curative. The present case describes a 63‐year‐old Vietnamese man with cardiovascular risk factors who was diagnosed with IMHMV after many months of severe symptoms. A review of the current literature follows the case report.

## Graphical abstract

Idiopathic myointimal hyperplasia of the mesenteric veins (IMHMV) is an inflammatory bowel disease‐mimic, characterised by intimal smooth muscle hyperplasia resulting in thickened small and medium‐sized mesenteric veins (with arterial‐sparing). We describe a 63‐year‐old Vietnamese man with cardiovascular risk factors who was diagnosed with IMHMV after many months of severe symptoms. A review of the current literature, including 33 published cases of IMHMV, follows the case report.

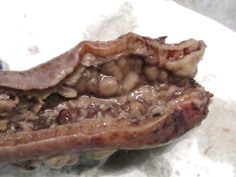

## Case presentation

A 63‐year‐old Vietnamese man was electively admitted to a tertiary hospital in Melbourne after undergoing an outpatient colonoscopy to investigate progressive bloody diarrhea. Symptoms over the 3 months preceding admission included acute‐onset watery diarrhea, up to 10 bowel motions per day, fecal incontinence, and significant weight loss. There was no associated pain, nausea, or infective symptoms. In the community, a trial of metronidazole and an antidiarrheal agent was ineffective. Past medical history included a basal ganglia hemorrhage with residual left‐sided weakness and hypercholesterolemia, and he took regular medications for secondary stroke prevention. He had no family history of bowel cancer or inflammatory bowel disease (IBD). Apart from antibiotic exposure he did not have infective risk factors such as recent overseas travel or sick contacts.

**Figure 1:**
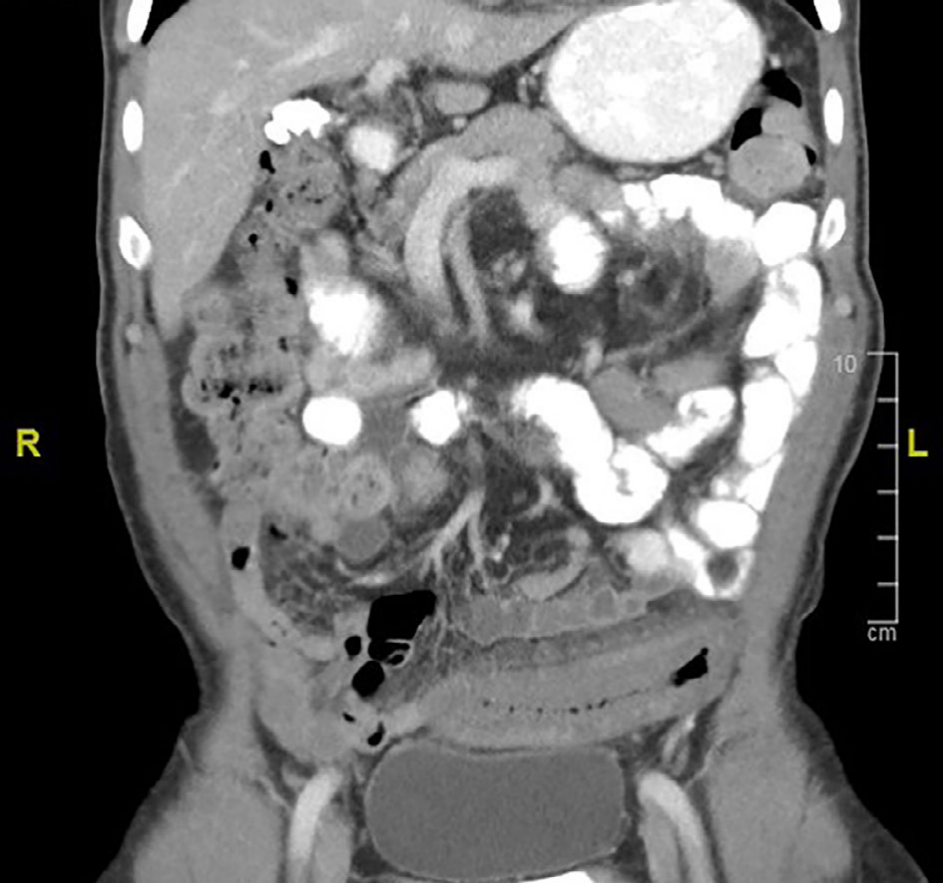
Computed topography (CT) abdomen (coronal view) showing contiguous concentric thickening of the distal descending colon to the rectum with stranding of the adjacent colonic fat.

On examination, the patient was afebrile; his heart rate was 84 beats/min, and body mass index was 22.4 kg/m^2^, having lost 14 % of his body weight in 4 months. Abdominal examination demonstrated a soft abdomen, with no tenderness or peritonism. Severe malnutrition was present with loss of muscle mass and subcutaneous fat. There was no lymphadenopathy or clinical signs of thyrotoxicosis. Per‐rectal examination was unremarkable. Laboratory investigations showed mild lymphocytopenia (1.1 × 10^9^/L) but an otherwise unremarkable routine blood panel. A nutrition screen showed hypoalbuminemia (albumin 32 g/L). A septic screen was negative, including QuantiFERON‐TB Gold and human immunodeficiency virus serology. There were moderate numbers of leukocytes and erythrocytes on a fecal specimen but no cultured bacteria and parasites, and *Clostridium difficile* toxin was not detected. Fecal calprotectin was mildly elevated (96.5 μg/g). Abdominal computed topography (CT) indicated thickening of the distal descending colon, favoring an infective or inflammatory colitis (Fig. 1).

**Figure 2:**
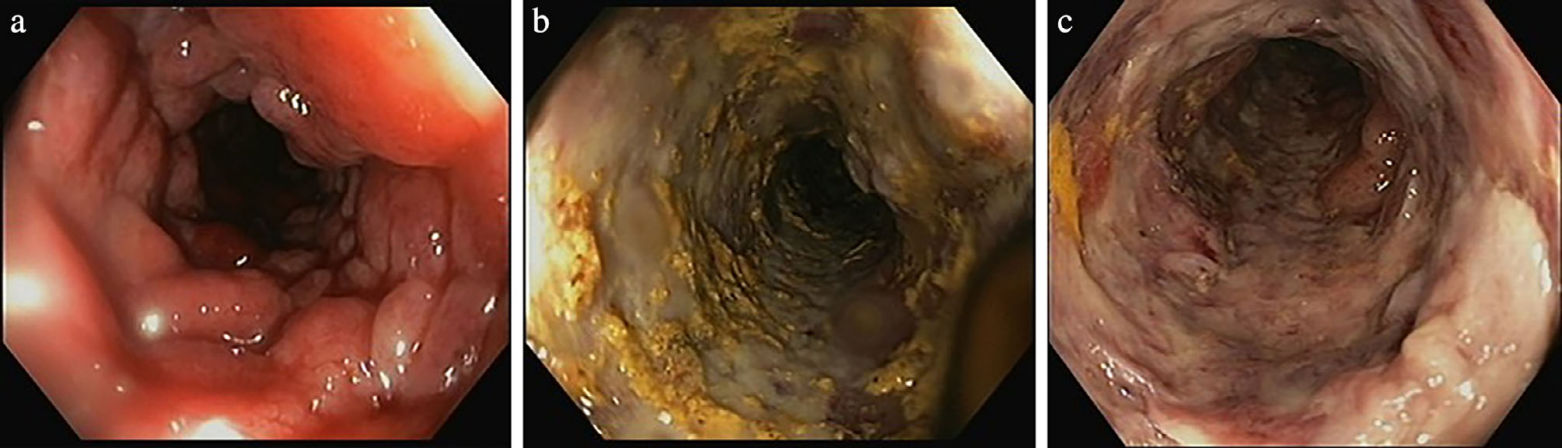
(a–c) Endoscopic images of sigmoid colon showing cobblestone mucosa (1a, 1c) and ulceration with exudates (1b).

Initial sigmoidoscopy demonstrated severely inflamed mucosa with cobblestone appearance of the sigmoid colon and well‐demarcated rectal sparing (Fig. 2a), although the rectum had lost its regular vascular pattern and had a scarred appearance. Histopathology was suggestive of ischemia with no evidence of dysplasia or malignancy. This prompted admission to hospital in the Gastroenterology unit. An attempted complete colonoscopy showed similar features extending proximally (Fig. 2b,c), and in the setting of severe inflammation, the procedure was abandoned to avoid risk of perforation. Biopsies of the descending colon demonstrated severe ulceration and granulation tissue and no amyloid deposition on Congo red stains. Viral and G*iardia*, C*ryptosporidium*, and *Entamoeba* polymerase chain reaction testing were negative, and there were no acid‐fast bacilli detected nor was there growth of mycobacterium. A vasculitic screen was negative on laboratory testing, and a CT‐angiogram demonstrated extensive colitis with dilatation of the transverse and ascending colon but no arteriopathy or evidence of mesenteric thrombus, although there was engorgement of the vessels primarily in the distal descending colon. Empirical treatment included intravenous antibiotics, corticosteroids, and total parenteral nutrition.

**Figure 3:**
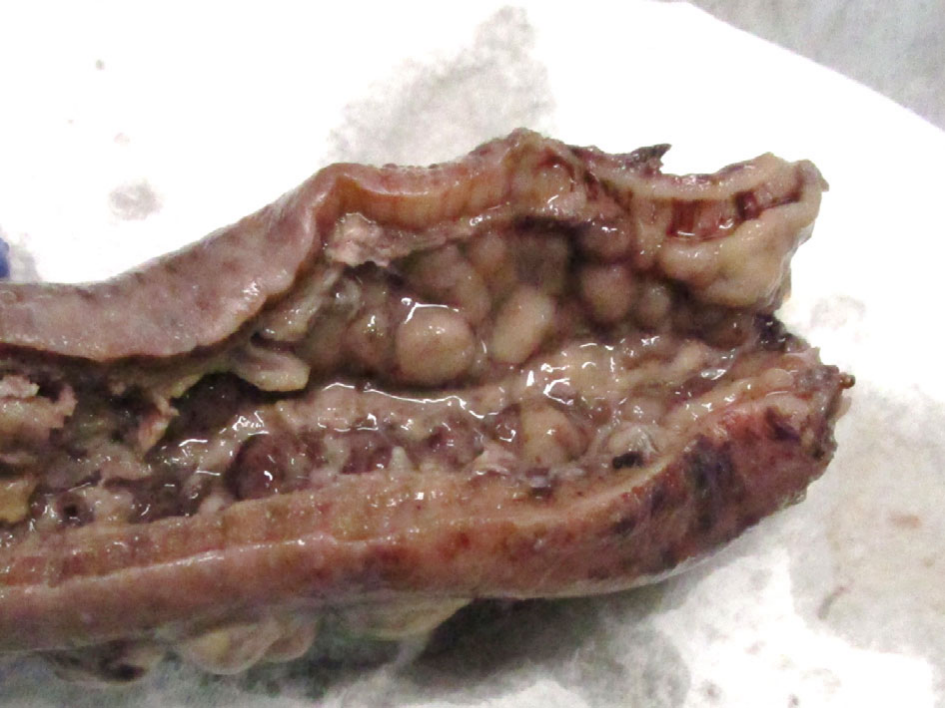
Macroscopic image of resected segment after fixation showing thickened bowel wall and cobblestone mucosa.

**Figure 4:**
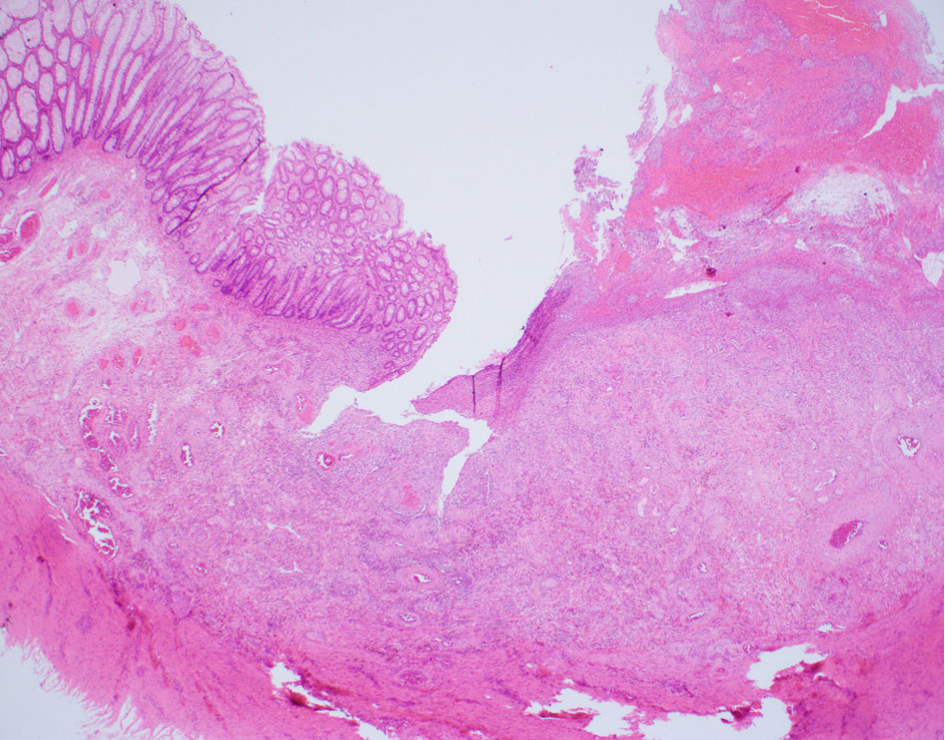
Microscopic image of resected colon ulcer with undermined features and numerous thick‐walled vessels in the submucosa. Hematoxylin and eosin. Magnification ×20.

**Figure 5:**
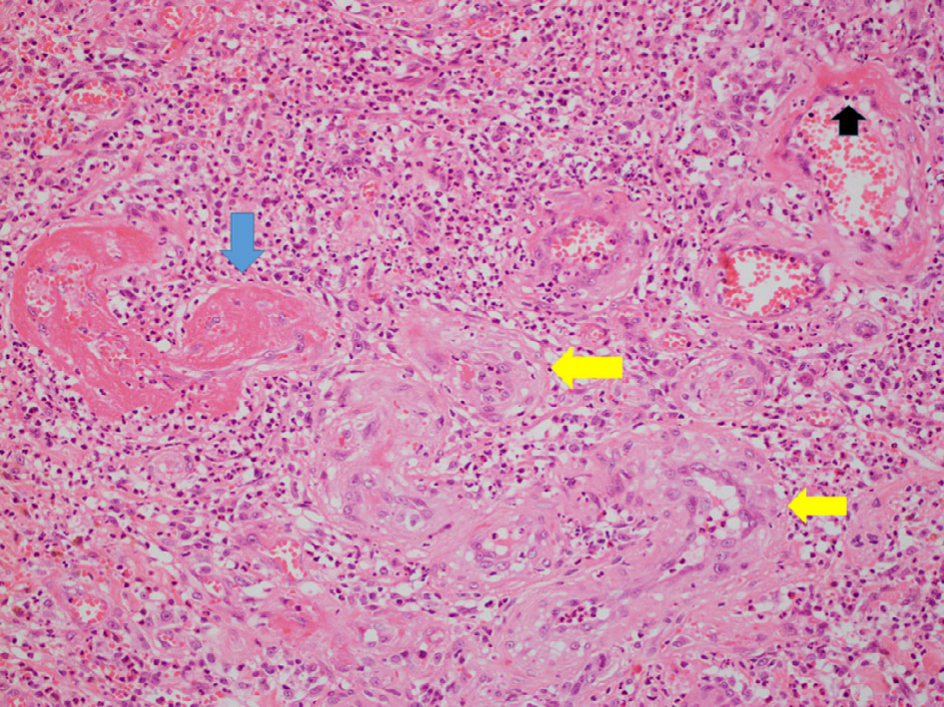
Medium‐power image of submucosa showing fibrin thrombi in vessels, (blue arrows), subendothelial fibrin deposits (black arrows), and arteriolization of capillaries (yellow arrows). Hematoxylin and eosin. Magnification ×200.

**Figure 6:**
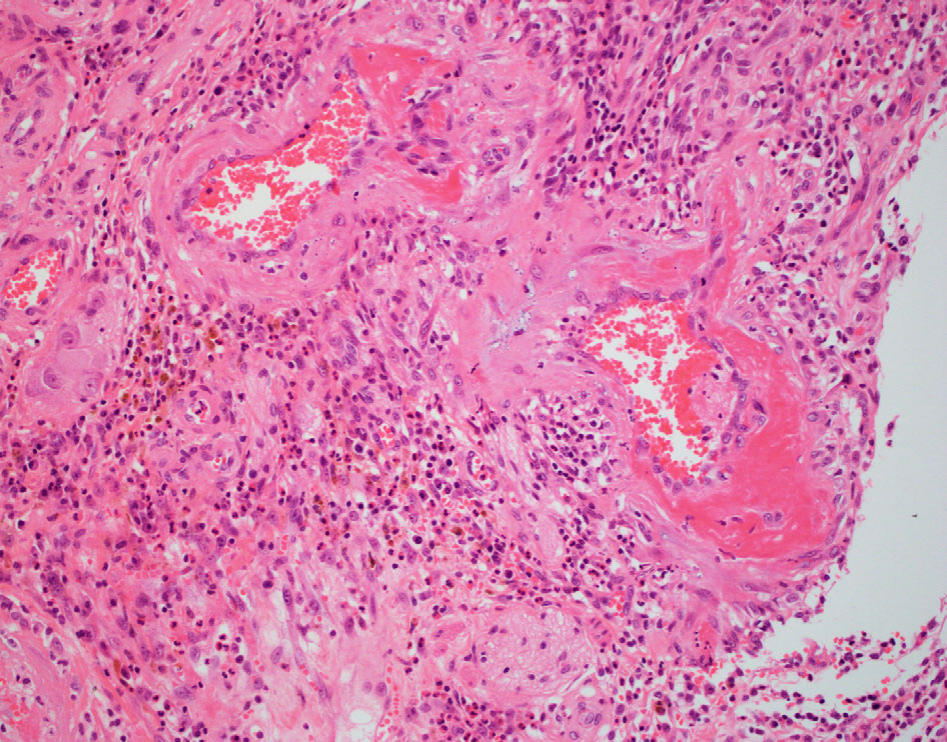
High‐power image of submucosa showing prominent subendothelial fibrin deposits and thick‐walled small vessels. Hematoxylin and eosin. Magnification ×400.

**Figure 7:**
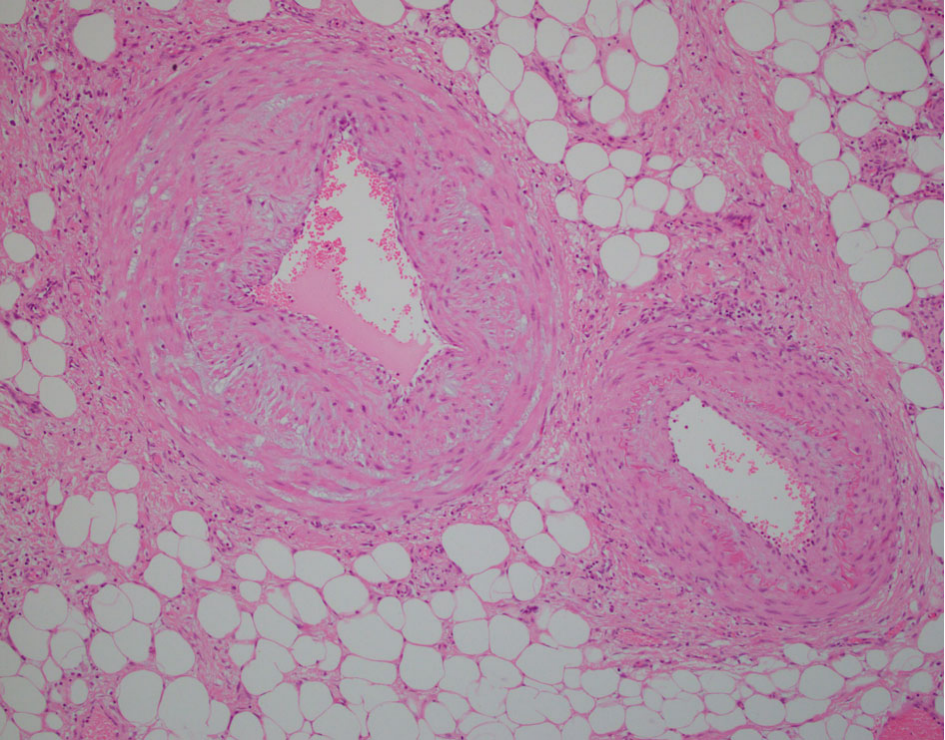
Microscopic image of mesenteric vein on left and mesenteric artery on right showing marked myointimal thickening with myxoid change and narrowing of the lumen in the vein. Hematoxylin and eosin. Magnification ×100.

**Figure 8:**
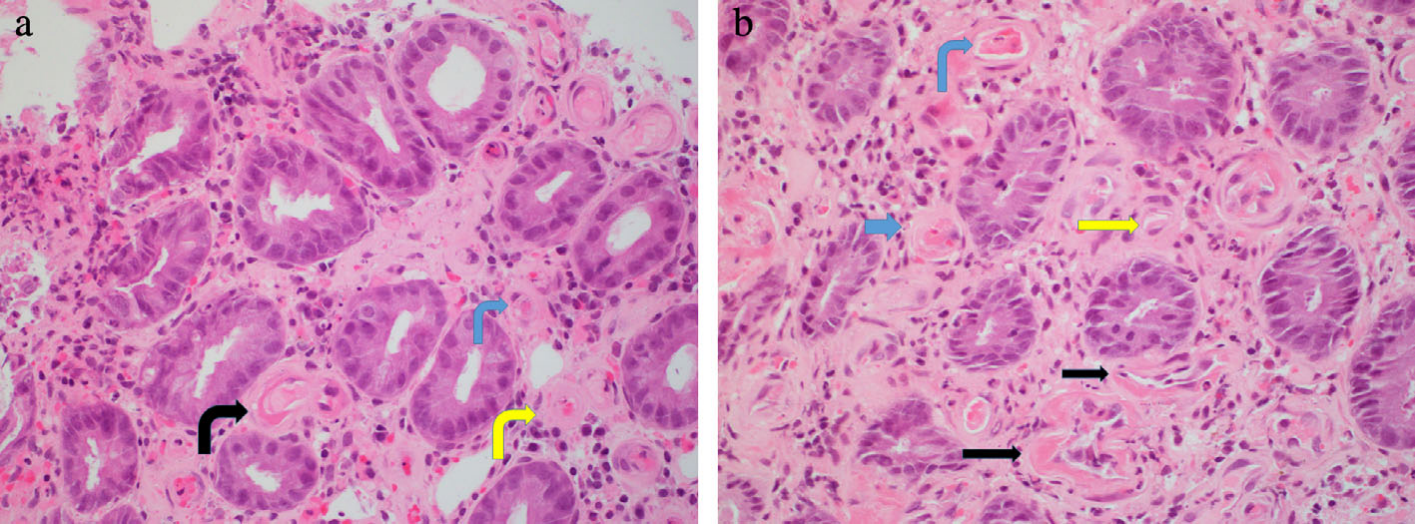
High power of (a) first series and (b) second series of endoscopic mucosal biopsies taken before surgery showing fibrin thrombi in vessels (blue arrows), subendothelial fibrin deposits (black arrows), and arteriolization of capillaries (yellow arrows). Hematoxylin and eosin. Magnification ×400.

Failure to respond to 3 weeks of medical therapy resulted in a multidisciplinary team decision to proceed with an open Hartmann's procedure. Intraoperative findings included a grossly abnormal colon from the upper rectum to mid‐descending colon. The rectum and mesentery were edematous, and the affected bowel mesentery was adherent and fixed to the retroperitoneum. There were no signs of any other disease, and blood supply to the bowel was normal. Macroscopic examination of the bowel showed diffusely thickened wall, with thickened mesenteric fat and extensive areas of ulceration (Fig. 3). Histopathology of the sigmoid colon showed extensive ulceration of the bowel wall, including submucosa (Fig. 4). There were extensive ischemic changes with crypt atrophy and attempted regeneration and mucosal hemorrhage. Distinctive features at the base of the ulcer, the submucosa, and even in areas with intact mucosa were: (i) capillaries with marked mural fibrous thickening, so called “arteriolization” of capillaries; (ii) focal subendothelial deposits of fibrin in small vessels); and (iii) fibrin thrombi in small vessels (Figs 5 and 6). The large veins in the mesenteric fat and in the subserosal fat showed prominent myxoid change with myointimal hyperplasia, causing prominent narrowing of the lumen (Fig. 7). These veins often appeared larger than the associated arteries. One vein showed evidence of recanalization consistent with previous thrombosis. These changes were those of ischemic colitis associated with myointimal hyperplasia of mesenteric veins. The three sets of mucosal biopsies taken in the 7 weeks prior to surgery were reviewed, and all showed, in addition to the changes of ischemic colitis, the characteristic mucosal changes of this disorder—fibrin thrombi and subendothelial fibrin deposits in small vessels and the arteriolization of small vessels, which are not seen in other causes of ischemic colitis (Fig. 8a,b).

The patient made good postoperative recovery and was discharged home 2 weeks later, tolerating an oral diet and managing his stoma independently.

## Discussion

### *Review of the literature*

Idiopathic myointimal hyperplasia is a rare cause of intestinal ischemia. Unlike its counterparts, idiopathic myointimal hyperplasia of the mesenteric veins (IMHMV) is not caused by arterial thromboembolism, venous thrombus or vasculitis.^1^ The etiology of this nonthrombotic occlusion of mesenteric veins is poorly understood. First described by Genta and Haggitt in 1991, 33 reported cases of IMHMV have now been reported (Table 1),^1^, ^2^, ^3^, ^4^, ^5^, ^6^, ^7^, ^8^, ^9^, ^10^, ^11^, ^12^, ^13^, ^14^, ^15^, ^16^, ^17^, ^18^, ^19^, ^20^, ^21^ and it is likely there are many more misdiagnosed or not reported.

**Table 1:** Reported cases of IMHMV: clinical features

Case	Authors, year	Age/gender	CVRFs	Location	Clinical diagnosis	Indication for surgery	Time to surgery	Follow up
1	Martin *et al*., 2018	63M	Y	RS	IC/IBD	Persisting symptoms	5 months	2 months
2	Gonai *et al*., 2016	68M	—	DC + S	Mesenteric panniculitis	Diagnosis on biopsy/persisting symptoms	—	—
3	Song & Shroff, 2016	59M	N	RS	IBD	Persisting symptoms	30 years	2 weeks
4	Yantiss *et al*., 2017	71M	—	DC + R	IC/IBD	—	—	—
5	Yantiss *et al*., 2017	83M	—	—	IC	—	—	—
6	Yantiss *et al*., 2017	83M	—	DC + R	IBD	—	—	—
7	Yantiss *et al*., 2017	63M	—	DC + R	IBD	—	—	—
8	Yantiss *et al*., 2017	78M	—	DC + R	IBD	—	—	—
9	Yantiss *et al*., 2017	73F	—	DC	IBD	—	—	—
10	Yantiss *et al*., 2017	65M	—	DC + R	IBD	—	—	—
11	Yantiss *et al*., 2017	64M	—	DC + R	IBD	—	—	—
12	Yantiss *et al*., 2017	25M	—	S	IC	—	—	—
13	Yantiss *et al*., 2017	71M	—	DC + R	IBD	—	—	—
14	Cauchois *et al*., 2016	48M	—	—	IBD/digestive vasculitis	—	—	—
15	Patel *et al*., 2016	65M	N	RS	IBD	Perforation	1.5 months	—
16	Yang *et al*., 2016	44M	N	RS	IBD	Persisting symptoms	1 months	—
17	Yun *et al*., 2016	64M	Y	RS	IBD	Hematochezia	2 years	6 months
18	Costa *et al*., 2016	47M	—	RS	—	Persisting symptoms	9 months	—
19	Abbott *et al*., 2015	58M	—	DC + R	IC/IBD/infection	Persisting symptoms	—	—
20	Sahara *et al*., 2015	76M	Y	RS	IC/IBD/infection	Pain	12mo	3 months
21	Wangensteen *et al*., 2015	62F	Y	RS	IBD	Pain	2 months	1.5 years
22	Laskaratos *et al*., 2015	62F	N	Ileal	—	Perforation	—	—
23	Zijlstra *et al*., 2014	62M	—	—	—	Acute abdomen	—	—
24	Korenblit *et al*., 2014	59M	—	RS	IC	Persisting symptoms	>1 months	9 months
25	Korenblit *et al*., 2012	62M	Y	RS	IBD	Persisting symptoms	>1 years	—
26	Feo *et al*., 2013	75F	—	RS	—	Persisting symptoms	6 months	—
27	Lanitis *et al*., 2012	81M	N	Terminal ileum	—	AM + PP	6 months	—
28	Chiang *et al*., 2012	60M	—	RS	IBD	—	2 months	4 months
29	Garcia‐Castellanos *et al*., 2011	32F	Y	RS	PPI/pseudomembranous colitis	Pain + bleeding	3 months	2 years
30	Kao *et al*., 2005	38M	N	RS	Idiopathic IBD	Perforation	5 months	1.5 years
31	Savoie & Abrams, 1999	22M	—	RS	Idiopathic proctosigmoiditis/IBD	Bleeding	—	10 months
32	Bryant, 1998	42F	Y	Jejunum	—	—	—	RIP
33	Abu‐Alfa *et al*., 1996	58M	N	S	IC/IBD	Pain + bleeding	1 years	—
34	Genta & Haggitt, 1991	30M	N	S	Stricture	Bowel obstruction	1 months	7 years
35	Genta & Haggitt, 1991	38M	—	DC + R	IBD	Toxic megacolon	2 months	1 years
36	Genta & Haggitt, 1991	25M	N	RS	IBD	Acute abdomen	>6 months	4 years
37	Genta & Haggitt, 1991	67M	N	S	IBD	Persisting symptoms	3 months	2 years

AM, appendiceal mucocoele; CVRFs, cardiovascular risk factors; DC, descending colon; IBD, inflammatory bowel disease; IC, ischemic colitis; PP, *Pseudomyxoma peritonei*; PPI, primary pneumatosis intestinalis; R, rectum; RS, rectosigmoid; S, sigmoid.

Of the 34 cases of IMHMV that occurred in men, usually in late middle age (median age at diagnosis 62 years).^1^, ^2^, ^3^, ^4^, ^5^, ^6^, ^7^, ^8^, ^9^, ^10^, ^11^, ^12^, ^13^, ^14^, ^15^, ^16^, ^17^, ^18^, ^19^, ^20^, ^21^ Many of these patients, including our patient, have cardiovascular risk factors or disease.^5^, ^8^, ^11^, ^12^, ^15^ The non‐specific presenting symptoms include diarrhea, usually bloody; lower abdominal pain; and weight loss, resulting in the frequent misdiagnosis of IBD. Patients are often treated with corticosteroids or other IBD therapies such as 5‐aminosalicylates, immunomodulators, or biologic agents.^1^, ^2^, ^4^, ^6^, ^7^, ^8^, ^11^, ^12^, ^13^, ^14^, ^15^, ^16^, ^17^

The most common site of disease is the left colon, particularly rectosigmoid colon. Three cases were reported to occur in the small bowel,^22^, ^23^, ^24^ but on careful review of these cases, these were excluded from the present series. All of the other cases involved the left colon. The first case of jejunal involvement^22^ showed only one vein rather than multiple with eccentric luminal occlusion by what looks like inflamed organized thrombus rather than fibromxyoid spindle cell proliferation in the intima. Sherman and colleagues^25^ have highlighted that small focal lesions in mesenteric veins resembling IMHMV but distinct from the more diffuse lesions in classical IMHMV can be found in bowel associated with preresection healed trauma, including volvulus, intussusception, incarcerated hernia; stoma takedown; or anastomoses in the involved bowel segment, as well as colonic resections for diverticulitis and inflammatory bowel disease. Kao *et al*.^4^ also noted the finding of focal IMHMV lesions in bowel segments removed for diverticulitis. The second case of terminal ileum^23^ involved bowel intimately associated with neoplastic pseudomyxoma peritonei and a perforated low‐grade appendiceal mucinous neoplasm. Mucosal changes typical of IMHMV were not described, and the image of purported lamina propria vessels showed serosal vessels and muscularis propria. The third case of small bowel perforation occurred in a patient with a complex medical history, including primary biliary cirrhosis and renal transplantation secondary to reflux nephropathy.^24^ The article format of photographic snapshot precluded detailed clinical information, such as the patient's medication history and histological description. Mesenteric vein changes as seen in IMHMV were depicted, but no details of the distribution of these changes throughout the small bowel and right colon or typical mucosal features of thick‐walled small vessels and subendothelial fibrin were reported. This patient also suffered recurrent ulceration at the anastomotic site after right hemicolectomy.

The radiological appearance of IMHMV on computed tomography (CT) is suggestive of colitis, with thick, edematous bowel and fat stranding.^4^, ^5^, ^9^, ^12^, ^13^, ^14^, ^15^, ^17^, ^20^, ^21^ Strictures in the sigmoid colon have also been reported.^1^ No vascular occlusions should be identified on CT‐angiography.^6^, ^10^, ^13^ Common endoscopic findings include luminal narrowing due to mucosal edema and congestion. The inflamed friable mucosa leads to significant ulceration.^19^ In the majority of cases, initial investigations, including endoscopy and histological review, led to a suspected diagnosis of ischemic colitis.^1^, ^4^, ^7^, ^9^, ^10^, ^11^, ^12^, ^13^, ^14^

In three cases, the diagnosis of IMHMV was reached prior to surgical resection via endoscopy and histology.^11^, ^17^, ^21^ Most patients underwent surgical resection due to significant morbidity and a lack of improvement with corticosteroids or other IBD treatments. Two patients required urgent surgery due to rectosigmoid perforation,^4^, ^17^ and a further four had either acute abdomen or bowel obstruction prompting surgery.^1^, ^10^ The median time between symptom onset and surgery was 5 months.^1^, ^2^, ^3^, ^4^, ^5^, ^6^, ^7^, ^8^, ^9^, ^10^, ^11^, ^12^, ^13^, ^14^, ^15^, ^16^, ^17^, ^18^, ^19^, ^20^, ^21^ Postoperative recurrence has not been reported, with a follow‐up duration of up to 7 years.^1^

### *Pathogenesis*

The pathogenesis of the myointimal hyperplasia in mesenteric veins is unknown. Genta and Haggitt and Abu‐Alfa and colleagues both noted that the venous changes were similar to those seen in saphenous vein graft failing around 12 months following coronary artery bypass surgery.^1^, ^2^ This has been attributed to two factors: (i) graft adaptation to higher arterial pressure and (ii) loss of inhibition from endothelium due to mechanical trauma and distension of the walls.^26^ Several authors have suggested the development of an arteriovenous fistula to produce arterial‐level pressure in the veins, but no cases have been demonstrated to confirm this theory.^1^, ^2^, ^4^ The striking left‐sided colonic involvement is likely to have a significant role in the pathogenesis. The sigmoid colon is much more mobile than other areas of the colon, so torsion of the sigmoid mesocolon could result in stretching of veins and injury. Yantiss *et al*. have suggested that chronic mechanical stress from intermittent torsion on the veins of the rectosigmoid colon may play a role in the development of myointimal hyperplasia.^19^ These veins could then become narrowed and could thrombose with variable recanalization, and there would be resultant mucosal and submucosal ischemic changes. The venous hypertension in the wall of the affected colon is postulated to cause endothelial damage with fibrin deposition in the walls of capillaries and progressive thickening and arteriolization of the capillaries.

Another hypothesis is that IMHMV represents a burned out stage of enterocolic lymphocytic colitis as one case^27^ has been described with myointimal hyperplasia and phlebitis. This appears less likely as none of the cases in the literature report inflammation in the mesenteric veins.

### *Pathological discussion*

Although this entity was first described in 1991, it is still not recognized and diagnosed by gastroenterologists and anatomical pathologists until resection of the affected colonic segment. IMHMV does share some histological features of ischemic colitis unrelated to IMHMV—surface epithelial degeneration, crypt withering, and lamina propria hemorrhage. However, a recent, very elegant study by Yantiss and colleagues highlighted the pathognomonic three small vessel changes seen in mucosal biopsies and resections, “arteriolization” of capillaries, subendothelial fibrin deposits, and fibrin thrombi, which were typical of ischemic colitis due to IMHMV.^19^ These changes were not seen in other cases of ischemic colitis that were not associated with IMHMV, radiation colopathy, amyloidosis, small vessel vasculitis, diverticulitis, or Crohn's disease, apart from fibrin thrombi in 20 % of vasculitis cases and 10 % of ischemic colitis patients, but without the other two typical changes. Once the pathologist is aware of these characteristic features, the diagnosis can be made on endoscopic biopsies.

IMHMV is a rare but important diagnosis that leads to significant morbidity and mortality due to delayed diagnosis. Although the optimal medical treatment is unknown, prompt diagnosis and surgical resection is curative and can prevent prolonged symptoms, malnutrition, and complications such as bowel perforation. We aim to highlight the importance of IMHMV as a rare but increasingly recognized diagnosis of ischemic colitis without apparent arterial compromise.
